# Characterizing Croatian Wheat Germplasm Diversity and Structure in a European Context by DArT Markers

**DOI:** 10.3389/fpls.2016.00184

**Published:** 2016-02-22

**Authors:** Dario Novoselović, Alison R. Bentley, Ruđer Šimek, Krešimir Dvojković, Mark E. Sorrells, Nicolas Gosman, Richard Horsnell, Georg Drezner, Zlatko Šatović

**Affiliations:** ^1^Department for Breeding & Genetics of Small Cereal Crops, Agricultural Institute OsijekOsijek, Croatia; ^2^Centre of Excellence for Biodiversity and Molecular Plant BreedingZagreb, Croatia; ^3^The John Bingham Laboratory, National Institute of Agricultural BotanyCambridge, UK; ^4^Department of Plant Breeding and Genetics, Cornell University, IthacaNY, USA; ^5^Bayer Crop Science NVGhent, Belgium; ^6^Faculty of Agriculture, University of ZagrebZagreb, Croatia

**Keywords:** genetic diversity, population structure, AMOVA, wheat, DArTs, TriticeaeGenome

## Abstract

Narrowing the genetic base available for future genetic progress is a major concern to plant breeders. In order to avoid this, strategies to characterize and protect genetic diversity in regional breeding pools are required. In this study, 89 winter wheat cultivars released in Croatia between 1936 and 2006 were genotyped using 1,229 DArT (diversity array technology) markers to assess the diversity and population structure. In order to place Croatian breeding pool (CBP) in a European context, Croatian wheat cultivars were compared to 523 European cultivars from seven countries using a total of 166 common DArT markers. The results show higher genetic diversity in the wheat breeding pool from Central Europe (CE) as compared to that from Northern and Western European (NWE) countries. The most of the genetic diversity was attributable to the differences among cultivars within countries. When the geographical criterion (CE vs. NWE) was applied, highly significant difference between regions was obtained that accounted for 16.19% of the total variance, revealing that the CBP represents genetic variation not currently captured in elite European wheat. The current study emphasizes the important contribution made by plant breeders to maintaining wheat genetic diversity and suggests that regional breeding is essential to the maintenance of this diversity. The usefulness of open-access wheat datasets is also highlighted.

## Introduction

Common wheat (*Triticum aestivum*) is an allohexaploid, combining the genomes of three ancestral diploid grass species, the A-genome of *Triticum urartu*, the B-genome from a species related to *Aegilops speltoides* and the D-genome of *Aegilops tauschii* (Dvorak and Zhang, 1992). The allopolyploid nature and origin of common wheat undoubtedly contributes to its adaptability since its progenitors grow in a wide range of environments from the southern coast of the Caspian Sea, across northern Iran, Turkmenistan, and northern Afghanistan to China (Zohary et al., 1969).

Historically, domestication was the first bottleneck in reducing genetic variation in many crops (Morgante and Salamini, 2003). In wheat the bottleneck was accentuated further as the interspecific crosses that gave rise to hexaploid wheat occured only a few times (a founder effect). Furthermore, early farmer selection caused genetic drift and depletion of certain alleles from a gene pool (Cox, 1998). A second bottleneck was caused by the post-Mendelian adoption of breeding procedures separating environmental from genetic effects (Morgante and Salamini, 2003) and contributing to the depletion and reduction of diversity by replacing local landraces with newly improved varieties (Harlan, 1972).

A major concern for plant breeders is the narrowing of the genetic base of breeding material which lowers the likelihood of genetic progress. van de Wouw et al. (2010) conducted a meta-analysis of genetic diversity studies in the 20th century for different crop varieties, suggesting there were no clear general trends in genetic diversity for crop varieties released in the last century. What their study revealed was a significant reduction in the diversity of released varieties in the 1960s, but even then the diversity reduction as compared with the diversity levels in the 1950s was only 6%.

There are several issues to be addresed in monitoring genetic diversity.

It is important to identify the initial replacement of landraces by modern varieties, to assess the impact of selective breeding on genetic erosion caused by replacing unique with common alleles and accounting for the influence of seed companies releasing similar varieties in different regions.

Furthermore, it has been observed that limited breeding activity leads to less diversity narrowed by the number of released varieties, which is a possible threat to farmer’s or seed producer’s ultimate choice of varieties (van de Wouw et al., 2010).

Population structure analysis also provides deeper understanding of genetic diversity in a given germplasm set and the necessary information for association mapping in which accurate estimates of population structure are needed for the control of relatedness in mixed-model association mapping studies (Yu et al., 2006; Zhu et al., 2008). Bayesian model-based clustering, which models variation in ancestral subpopulations along a chromosome as a Markov process (Astle and Balding, 2009), assigns an individual to one of K populations based on the information of its genotype and information about the distribution of the various alleles in K populations (Pritchard et al., 2000) providing insight about gene flow patterns and migration rates (Guillot and Carpentier-Skandalis, 2011).

Because of the all above-mentioned, it is important to have available reliable tools for monitoring and measuring genetic diversity and population structure.

The assessment of genetic diversity relied on the use of pedigree information, morphological (passport data) and biochemical (isozymes and storage proteins) markers (Mohammadi and Prasanna, 2003). With these approaches, genetic diversity among major field crops such as bread wheat (Cox et al., 1986; Souza et al., 1994), durum wheat (Autrique et al., 1996), maize (Smith and Smith, 1987, 1988), barley (Martin et al., 1991), and soybean (Cardy and Beversdorf, 1984; Cox et al., 1985) was described. These assessments based on pedigree information are sufficiently reliable (when data about parents are correct), but sometimes they do not mirror accurately parentage and do not take into account the effects of selection, mutation and genetic drift. In contrast, DNA markers allow the assessment of genetic relationship at the DNA level directly (Laidò et al., 2013).

In the past two decades there was an explosion of different types of DNA markers (Queen et al., 2004; Landjeva et al., 2006) such as: (1) restriction fragment length polymorphisms (RFLPs); (2) random amplified polymorphic DNAs (RAPDs); (3) amplified fragment length polymorphisms (AFLPs); (4) microsatellites or simple sequence repeats (SSRs); (4) plant retrotransposon (LTR based markers); (5) single nucleotide polymorphisms (SNPs); and (6) diversity array technology markers (DArTs).

The most common type of DNA markers that have been used in assessing genetic diversity in wheat is microsatellite or SSRs markers. Microsatellites have been proposed as one of the most suitable markers for the assessment of genetic diversity among wheat accessions, because they are multi-allelic, abundant, chromosome specific and evenly distributed along chromosomes (Röder et al., 1995). The major drawback of microsatellites is they require to be isolated *de novo* for each species (or a group of closely related species), because they are mostly located in non-coding regions where the nucleotide substitution rate is higher than in coding regions (Zane et al., 2002).

Since this time, marker systems have evolved to include DArT markers which assays for the presence (or amount) of a specific DNA fragment from the total genomic DNA, and simultaneously types several thousand loci in a single assay (Jaccoud et al., 2001). Although more advanced tools, such as SNP markers have become more widely used, Akbari et al. (2006) validated and demonstrated that DArTs perform very well in revealing the genetic relationship among bread wheat varieties and behave in a Mendelian fashion concluding that they can be effectively and informatively used to genotype polyploid species such as wheat.

The main objectives of this study were to use DArTs for (1) assessing genetic diversity available in the Croatian winter wheat breeding pool; (2) describing genetic population structure; (3) providing new information about the level of genetic diversity and structure from a single eco-geographic region; and (4) placing the genetic relationship in the Croatian breeding pool (CBP) in the wider context of European-wide winter wheat diversity as facilitated by combined, open-access DArT genotypes.

## Materials and Methods

### Germplasm

#### Croatian Breeding Pool (CBP)

A set of 89 winter wheat cultivars belonging to three different breeding programs [Agricultural Institute Osijek (PIO) (63), Bc Institute for Plant Breeding and Production of Field Crops Production, Zagreb (Bc) (23) and University of Zagreb, Faculty of Agriculture (FAZ) (3)] released in Croatia in the period 1936 to 2006 was selected to represent changes in breeding activities in the country over 70 years (**Supplementary Table S1**). The cultivars were bred in two regions: eastern Croatia (PIO) and western Croatia (Bc; FAZ).

#### European Breeding Pool (EBP)

In order to place the CBP in a European context, accessible DArT genotypes from two published winter wheat panels were included in this study. The first was the TriticeaeGenome (TG) panel: a panel of 376 elite wheat varieties from France, Germany and the UK (Bentley et al., 2014, dataset available at www.cerealsdb.uk.net). The second was a European diversity panel (ED) consisting of 94 mostly European wheat varieties recently described by Nielsen et al. (2014). A summary of the number of inbred lines in each panel, and across the panels, and the number of countries represented is given in **Supplementary Table S2**.

### DArT Genotyping

For DArT analysis of the CBP, DNA was extracted from young wheat leaf tissue from a single plant of each genotype using the protocol recommended by Triticarte Pty. Ltd. (http://www.triticarte.com.au/content/DNA-preparation.html) and sent to Triticarte for DArT analysis using common wheat PstI(TaqI) version 2.5 array. A total of 1531 DArT markers were scored, out of which 1,229 markers were retained, based on Q value (an estimate of marker quality) above 80, for further analysis.

Additional DArT data representing the EBP was collated from previous datasets (Bentley et al., 2014; Nielsen et al., 2014). There were 16 overlapping lines between the TG and ED panels and duplicates were retained where there were genotyping discrepancies (10 lines). Across the CBP and EBP only countries represented by at least 10 cultivars and common markers, removing those with ≥10% missing data, were used. The data was thinned by removing one marker from each pair of markers with an absolute correlation coefficient of >0.95 and a minor allele frequency of 0.05. The final dataset combining the CBP and EBP consisted of 523 lines from seven countries and 166 DArT markers (**Supplementary Table S2**). The 166 DArT markers were assigned map positions based on published mapping information (Huang et al., 2012).

### Data Analysis

#### Genetic Diversity of Croatian Winter Wheat Breeding Pool

Genetic diversity of Croatian winter wheat cultivars from two breeding programs (PIO and Bc/FAZ) as assessed using DArT markers was analyzed using the following parameters: percentage of polymorphic loci (%*P*), Shannon’s diversity index (*Sh*; Lewontin, 1972), effective number of alleles (*N_E_*; Berg and Hamrick, 1997), expected heterozygosity (*H_E_*; Nei, 1973), and polymorphic information content (*PIC*; Botstein et al., 1980).

In order to quantify rare markers in wheat cultivars from each program we calculated the modified ‘frequency down-weighted marker values (DW)’ based on the measure proposed by Schönswetter and Tribsch (2005) and implemented in AFLPdat (Ehrich, 2006). Instead of counting the number of occurrences of each marker in each group of cultivars and dividing it by the number of occurrences of that particular marker in the total dataset, we divided the frequency of each marker in each group of cultivars by the frequency of that particular marker in the total dataset. Finally, the rarity index (*RI*) for each program was calculated as average over all marker loci:

$${RI}_{j}=\frac{1}{I}\overset{I}{\underset{i=1}{\Sigma }}\frac{p_{ij}}{P_{i}}$$

where *I* is the number of markers, *p_ij_* is the frequency of *i*th marker in a group of cultivars *j* and *P_i_* is the frequency of ith marker in the total dataset. Thus, we even out the unequal sample sizes. The value of *RI* is expected to be higher for a program in which overall rare markers were frequent among cultivars.

The estimates of diversity parameters between cultivars were compared using repeated measures analysis of variance carried out using PROC GLM in SAS v. 9.2 (SAS Institute, 2004).

Genetic distances between pairs of cultivars were calculated by Dice’s distance coefficient (*D_Dice_*_;_ Dice, 1945). Cluster analysis based on dissimilarity matrix was performed using the Neighbor joining (NJ) method and the statistical support of the branches was tested with bootstrap analysis using 1,000 replicates (Felsenstein, 1985). The calculations were made using PAST version 2.01 (Hammer et al., 2001).

The analysis of molecular variance (AMOVA; Excoffier et al., 1992) using ARLEQUIN ver. 3.0 (Excoffier et al., 2005) was used to partition the molecular diversity based on DArT markers separately between and within two Croatian wheat breeding programs, namely PIO and Bc/FAZ. The variance components were tested statistically by non-parametric randomisation tests using 10,000 permutations.

A model-based clustering method was applied to infer genetic structure of Croatian winter wheat breeding pool using the software STRUCTURE ver. 2.3.3 (Pritchard et al., 2000). Ten runs per each cluster (*K*) ranging from 1 to 11 were carried out on the Isabella computer cluster at the University of Zagreb, University Computing Centre (SRCE). Each run consisted of a burn-in period of 200,000 steps followed by 10^6^ Monte Carlo Markov Chain (MCMC) replicates assuming an admixture model and correlated allele frequencies. The choice of the most likely number of clusters (*K*) was determined according to *ad hoc* statistic *ΔK*, as described by Evanno et al. (2005) and as implemented in Structure-sum Ver. 2011 (Ehrich et al., 2007).

The admixture was quantified using Shannon’s diversity index based on proportions of membership in each cluster inferred by STRUCTURE. In this sense, Shannon’s diversity index will be equal to zero for a cultivar having 100% of its genome estimated to belong to a cluster while it will reach its maximum value when the proportions of membership of a given cultivar are equal in all clusters (the most admixed cultivar). Furthermore, the total diversity (*Sh_Total_*) of a breeding program could be calculated from the mean proportions of membership and related to the mean within-cultivar diversity (*Sh_Mean_*) calculated by averaging the Shannon’s diversity indices of the cultivars belonging to a breeding program. Thus, the admixture level of a breeding program can be separated into the proportion of admixture attributable to within-cultivar (*Sh_Mean_*/*Sh_Total_*) and among-cultivar [(*Sh_Total_*–*Sh_Mean_*)/*Sh_Total_*] admixture.

#### Genetic Relationships Among Winter Wheat Cultivars from Different European Countries

Diversity of DArT markers among wheat cultivars from seven European countries (Croatia, Denmark, France, Germany, Hungary, Sweden, UK) has been assessed using the same parameters as above (% *P*, *Sh*, *N_E_*, *H_E_*, *PIC*). The estimates of diversity parameters among countries were compared as above. *Post hoc* Bonferroni’s adjustments were used to compare the means of diversity estimates among countries at significance level *P* < 0.05.

In order to graphically represent genetic relationships among wheat cultivars from seven European countries, a factorial correspondence analysis (FCA) was carried out using Genetix 4.05 (Belkhir et al., 2004).

The AMOVA using ARLEQUIN was used to partition the molecular diversity of wheat cultivars based on DArT markers (A) within and among seven European countries and (B) among regions (Central Europe vs. Northern and Western Europe), among countries within regions and within countries. The Central European region was comprised of Croatia and Hungary while the Northern and Western European region included the remaining countries. Pairwise comparisons examined with AMOVA resulted in ϕ*_ST_* values that are equivalent to the proportion of total variance that is partitioned between groups of cultivars originating from two different countries. To obtain a distance matrix, ϕ*_ST_* values were interpreted as the inter-country distance average (Román et al., 2001). A cluster analysis based on the ϕ*_ST_* matrix was performed using the UPGMA method, as above.

A model-based clustering method was applied to infer genetic structure of Croatian winter wheat breeding pool using the software STRUCTURE ver. 2.3.3 (Pritchard et al., 2000). The genetic structure of European wheat cultivars population structure was assessed using STRUCTURE, as above.

## Results

### Genetic Diversity and Population Structure of Croatian Winter Wheat Breeding Pool

A total of 1,229 polymorphic DArT markers were included in the diversity analysis of 89 Croatian wheat cultivars. The Shannon’s diversity index (*Sh*) used to quantify the genetic diversity was 0.78 for PIO and 0.76 for Bc/FAZ. The average effective number of alleles per locus (*N_E_*) was 1.63 and 1.61, the expected heterozygosity (*H_E_*) 0.36 and 0.35, with an average of 0.38, while the polymorphism information content (*PIC*) was 0.29 and 0.28, with an average of 0.30, were found for PIO and Bc/FAZ, respectively. The rarity index (*RI*) was 0.985 for PIO and 1.036 for Bc/FAZ. A slightly higher genetic diversity was found in the winter wheat breeding pool from eastern Croatia (PIO), while overall rare markers were found more frequently among cultivars from western Croatia (Bc/AFZ; **Table 1**).

**Table 1:** Genetic diversity of Croatian wheat cultivars from two breeding programs.

Program	*n*	%*P*	*Sh*	*N_E_*	*H_E_*	*PIC*	*RI*
PIO	63	98.94	0.780	1.625	0.364	0.291	0.985
Bc/FAZ	26	98.86	0.763	1.606	0.354	0.283	1.036
*P*			*0.022*	*0.052*	*0.016*	*0.013*	*<0.0001*
**Total**	89	100.00	0.802	1.641	0.375	0.299	-


*PIO – Agricultural Institute Osijek, Bc – Bc Institute for Plant Breeding and Production of Field Crops Production, Zagreb, FAZ – University of Zagreb, Faculty of Agriculture; *P* – *P*-value of the test for difference between programs.*

The average genetic distance (*D_Dice_*) estimated for DArTs was 0.27. Four indistinguishable pairs of cultivars were found (*D_Dice_* = 0.00: C02 Afrodita/C12 Dvanaesta; C43 Nada/C61 Ratarka; C44 Neretva/C65 Ruža; C47 Njivka/C59 Poljarka), all originating from the breeding program carried out at the Agricultural Institute Osijek (PIO). The highest genetic distance (*D_Dice_* = 0.49) was calculated between cultivars C31 Lana and C38 Marija bred by the Bc Institute for Plant Breeding and Production of Field Crops Production, Zagreb (Bc).

**FIGURE 1:**
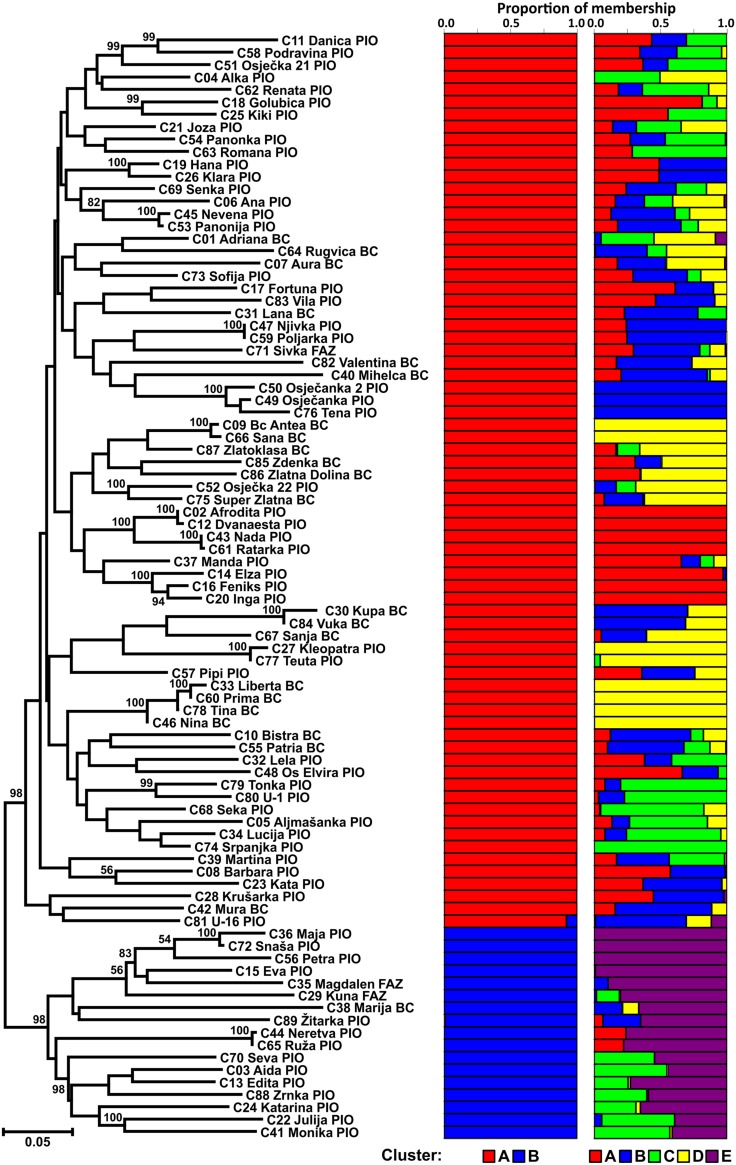
**Neighbor joining tree of 89 Croatian wheat cultivars and the proportion of membership in each cluster at *K* = 2 and 5 as defined with a model-based clustering method from Pritchard et al. (2000) based on 1,229 DArT markers**.

The Neighbor joining tree shows differentiation of the CBP into two clusters (**Figure 1**). The larger group included 72 cultivars of different origin (49 from PIO and 23 from Bc/AFZ) suggesting that breeders used the same or similar genetic material as parental lines. As a result, some cultivars from different breeding programs could show greater similarity than those from the same breeding program. The smaller group was comprised of 17 cultivars (14 from PIO and 3 from Bc/AFZ), that were mostly newly registered cultivars, except the cultivar Marija (Bc), which was one of the mostly widespread cultivars in the region during the 1980s and frequently used in crossings as a one of the parents.

Hierarchical analysis of genetic diversity using AMOVA was performed to analyze the partitioning of the genetic variation between and within breeding programs in CBP. Although most of the genetic diversity was attributable to differences among cultivars within a breeding program (94.36%), the significant ϕ*_ST_* values among breeding programs (ϕ*_ST_ = 0.06; P* < 0.0001) suggested the existence of moderate level of genetic differentiation between programs.

**FIGURE 2:**
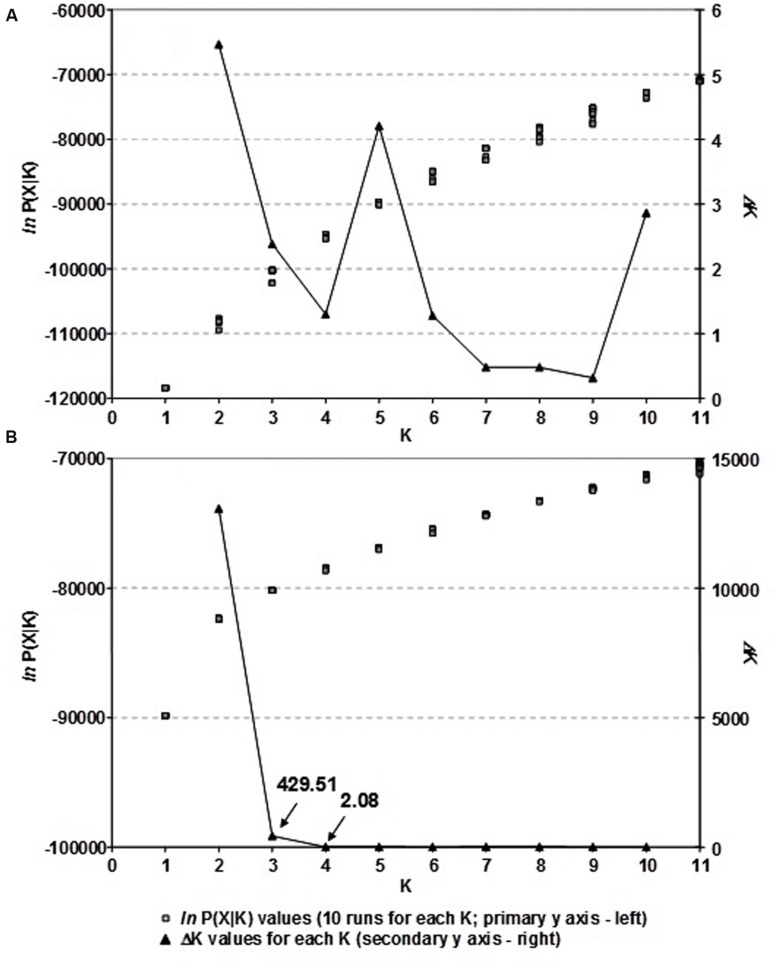
**(A)** The choice of the most likely number of clusters (K) inferred from 1,229 DArT markers of 89 Croatian wheat cultivars and **(B)** as inferred from 166 common DArT markers analyzing 523 wheat cultivars from seven European countries using a model-based clustering method of Pritchard et al. (2000): *ln P(X|* K*)* values for each of the ten independent runs for each K and *ΔK* values for each K based on the second order rate of change of the likelihood function with respect to K described by Evanno et al. (2005).

The population structure of Croatian wheat cultivars was assessed using the Bayesian model-based clustering method. In general, average estimates of the likelihood of the data, conditional on a given number of clusters, ln[Pr(X| K)], kept increasing with higher K (number of clusters), but the standard deviations among different runs for each *K* followed the same pattern (**Figure 2A**). The highest *ΔK* values were observed for *K* = 2 (5.47) followed by that at *K* = 5 (4.20). The proportion of membership of each Croatian wheat cultivar in each cluster at *K* = 2 and 5 is shown in **Figure 1**. At *K* = 2 the great majority of the cultivars were assigned to cluster A while the cluster B was comprised of 17 cultivars in complete concordance with the results of distance-based clustering analysis (**Figure 1**). All the cultivars had membership probabilities higher than 90% in a particular cluster. At *K* = 5 the majority of cultivars assigned to cluster B at *K* = 2 were included into cluster E.

The mean proportion of membership of Croatian wheat cultivars bred by the Agricultural Institute Osijek (PIO) and the Bc Institute for Plant Breeding and Production of field crops or the University of Zagreb, Faculty of Agriculture (Bc/FAZ) in each cluster at *K* = 5 is shown in **Supplementary Figure S1**.

The mean proportions of membership of PIO cultivars were in each of the five clusters ranged from 0.099 to 0.293. Bc/FAZ cultivars showed substantially higher proportions of membership in two clusters (D: 0.462; B: 0.290) while the proportions in the rest of the clusters was lower than 0.100. Shannon’s diversity index based on proportions of membership in each of the five clusters was used to assess the admixture levels of the Croatian breeding programs. Total Shannon’s diversity index of PIO cultivars (*Sh_Total_* = 0.674) was slightly higher than those of Bc/FAZ (*Sh_Total_* = 0.577) cultivars while the mean within-cultivar diversity of PIO cultivars (*Sh_Mean_* = 0.285) was lower than those of Bc/FAZ (*Sh_Mean_* = 0.301). Thus, the admixture attributable to within-cultivar component was lower than that attributable to among-cultivar component in case of PIO breeding program (42.31 vs. 57.69%, respectively) while considering Bc/FAZ breeding program the opposite was true (52.28 vs. 47.72%).

At *K* = 5 the number of cultivars classified as representative of a cluster (having more than 90% of their genome estimated to belong to a cluster) was 23, nine belonged to a cluster with membership probabilities between 75 and 90% while 57 could be considered as mixed (with membership probabilities < 75% for all clusters; **Supplementary Table S3**). Out of 26 cultivars bred by Bc/FAZ, six were classified as representative, all of them belonging to cluster D. On the other hand, seventeen out of 63 PIO cultivars classified as representative belong to all the detected clusters (A–E). The majority of cultivars from both programs were classified as mixed (Bc/FAZ: 69%; PIO: 62%).

### Genetic Diversity and Population Structure Among Winter Wheat Cultivars from Different European Countries

To contextualize the genetic diversity of the CBP, a total of 166 polymorphic DArT markers were included in the diversity analysis of 523 European wheat cultivars from seven countries (**Supplementary Table S2**). The 166 DArT markers in the thinned, combined CBP/EBP dataset were relatively evenly distributed across the bread wheat genome, although there were only 15 markers on the D genome, with no markers on either 4D or 6D (**Supplementary Table S4**). Using Shannon’s diversity index (*Sh*) it was found that genetic diversity varied from the highest levels found in Central European countries (Croatia: *Sh* = 0.83; Hungary: *Sh* = 0.73), toward the lowest, found in Nordic countries (Denmark: *Sh* = 0.61; Sweden: 0.60). The same pattern was observed for the expected heterozygosity (*H_E_*) and PIC. The highest number of effective alleles (*N_E_*) was found in Croatian and the lowest in the UK wheat pool (1.68 vs. 1.45). The rarity index (*RI*) value was 1.32 for Hungarian and 1.28 for Croatian wheat pool (**Table 2**).

**Table 2:** Genetic diversity of wheat cultivars from seven European countries based on 166 DArT markers (*n* = 523).

Country	*n*	%*P*	*Sh*	*N_E_*	*H_E_*	*PIC*	*RI*
Croatia	89	100.00	0.825 a^∗^	1.680 a	0.389 a	0.308 a	1.281 a
Denmark	22	86.14	0.607 cd	1.467 c	0.277 c	0.224 cd	0.923 b
France	214	100.00	0.677 bc	1.508 bc	0.306 bc	0.249 bcd	0.933 b
Germany	99	98.19	0.683 bc	1.526 bc	0.312 bc	0.251 bc	0.959 b
Hungary	11	89.16	0.724 b	1.586 b	0.340 b	0.270 b	1.323 a
Sweden	10	80.12	0.593 d	1.452 c	0.271 c	0.219 d	0.953 b
UK	78	95.78	0.610 cd	1.447 c	0.273 c	0.223 cd	0.900 b


*n – number of cultivars; %*P* – percentage of polymorphic markers; *Sh* – Shannon’s diversity index; *N_*E*_* – effective number of alleles; *H_*E*_* – expected heterozygosity; PIC – Polymorphic information content; *RI* – Rarity index.*

*^∗^Averages followed by the same letter are not significantly different among countries (*P* < 0.05).*

**FIGURE 3:**
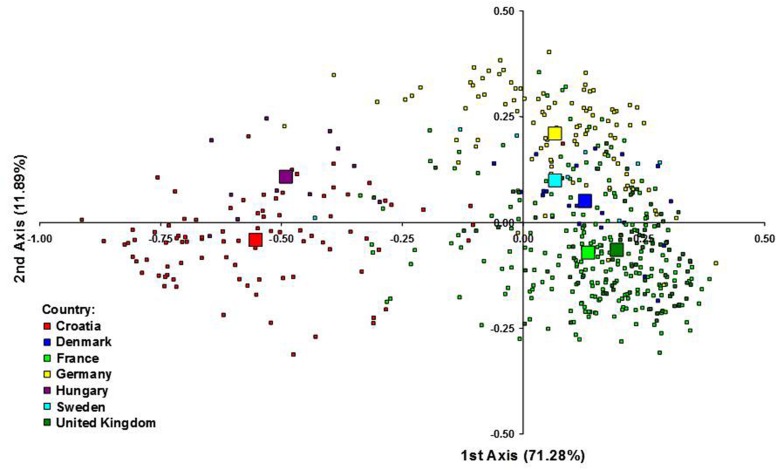
**Factorial correspondence analysis (FCA) of 523 wheat cultivars from seven European countries.** Each individual genotype is indicated by a small symbol, while the country barycentres are represented by larger ones.

**Figure 3** represents the genetic relationship among cultivars defined by the first two axes of the FCA, which accounted for 71.28 and 11.89% of the total inertia, respectively. Cultivars from CE countries clustered separately from the cultivars from NWE countries along the first axis, suggesting that the CE cultivars represented genetic diversity outside the NWE breeding pool. Along the second axis the cultivars from Germany and Sweden tended to be plotted separately from French and UK cultivars while the Danish cultivars were plotted in the middle position.

**FIGURE 4:**
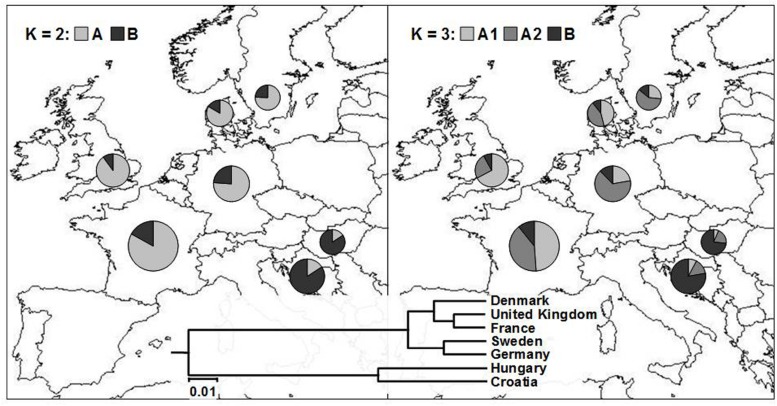
**Average proportion of membership of wheat cultivars in each of the seven European countries in each of the two (*K* = 2; left) and three (*K* = 3; right) clusters as defined with a model-based clustering method from Pritchard et al. (2000).** The UPGMA tree based on inter-country ϕ*_ST_* values is given at the bottom of the figure.

One-way AMOVA showed the most of the genetic diversity was attributable to the differences among cultivars within countries (88.69%; **Table 3**). ϕ*_ST_* value among countries was moderate, but highly significant (ϕ*_ST_ = 0.11; P* < 0.0001). When the geographical criterion (CE vs. NWE) was applied in the two-way AMOVA, highly significant difference between regions was obtained (ϕ*_ST_ = 0.16; P* < 0.0001) that accounted for 16.19% of the total variance while the differences among countries within regions accounted for only 3.50% (**Table 3**). Pairwise ϕ*_ST_* values among countries ranged from 0.021 between France and UK to 0.277 between UK and Hungary with the average value of 0.122 (**Table 4**). All the ϕ*_ST_* values were highly significant. Genetic differentiation between CBP and that of other countries was higher than average in all cases except in case of Hungary (ϕ*_ST_* = 0.074). As expected, the UPGMA tree based on inter-country ϕ*_ST_* values showed clear separation of CE cultivars from those belonging to NWE countries. In accordance with FCA results, the further subdivision between German and Swedish breeding pool from French, UK, and Danish cultivars was observed (**Figure 4**).

**Table 3:** Analysis of molecular variance for the partitioning of DArT diversity of wheat cultivars (A) among and within populations within CBP, (B) among and within countries, and (C) among regions (Central Europe vs. Northern and Western Europe), among countries within regions and within countries.

Analysis	Source of variation	df	Variance components	% Total variance	*ϕ*-statistic	*P(f)*
(A)	Among populations	1	0.00775	5.64	0.0536	<0.0001
	Within populations	87	0.12983	94.36		
(B)	Among countries	6	3.271	11.31	0.113	<0.0001
	Within countries	516	25.644	88.69		
(C)	Between regions	1	5.171	16.19	0.162	<0.0001
	Among countries within regions	5	1.119	3.50	0.042	<0.0001
	Among all countries	516	25.644	80.30	0.197	<0.0001


*Central Europe: Croatia, Hungary.*

*Northern and Western Europe: Germany, Denmark, France, UK, Sweden.*

**P*(ϕ) – ϕ-statistic probability level after 10,000 permutations.*

**Table 4:** Analysis of molecular variance (AMOVA’s) pairwise ϕ*_ST_* values (lower diagonal) and corresponding *P*-values (upper diagonal) between countries based on DArT markers.

Country	Croatia	Denmark	France	Germany	Hungary	Sweden	UK
Croatia		^∗∗∗^	^∗∗∗^	^∗∗∗^	^∗∗∗^	^∗∗∗^	^∗∗∗^
Denmark	0.176		^∗∗∗^	^∗∗∗^	^∗∗∗^	^∗∗^	^∗∗∗^
France	0.188	0.037		^∗∗∗^	^∗∗∗^	^∗∗∗^	^∗∗∗^
Germany	0.166	0.041	0.045		^∗∗∗^	^∗∗^	^∗∗∗^
Hungary	0.074	0.240	0.221	0.193		^∗∗∗^	^∗∗∗^
Sweden	0.145	0.052	0.049	0.029	0.213		^∗∗∗^
UK	0.219	0.034	0.021	0.064	0.277	0.071	


**P*-values as obtained by 10,000 permutations: “^∗∗∗^” corresponds to significance at the 0.1% nominal level and “^∗∗^” significance at the 1% nominal level.*

By analyzing the population structure of 523 cultivars from seven European countries using a Bayesian approach the similar pattern was observed, namely, that the average estimates of ln[Pr(X| K)] as well as the standard deviations among runs kept increasing with higher K (**Figure 2B**). The highest *ΔK* value were observed for *K* = 2 (13075.54) followed by that of *K* = 3 (429.51), while all the subsequent Ks had substantially lower *ΔK* values (<5). The clusters identified at *K* = 2 and *K* = 3 correspond well with those identified by FCA and UPGMA analysis based on pairwise ϕ*_ST_* values. At *K* = 2, the cluster A contained the majority of cultivars from NWE countries, while the majority of cultivars from CE countries belonged to cluster B (**Figure 4**). At *K* = 3, the cluster A was split into two subclusters (A1 and A2).

The most of the UK cultivars were assigned to the subcluster A1, the subcluster A2 comprised most of the cultivars from Germany and Sweden, while Danish and French cultivars were almost equally distributed among the two subclusters. The average proportion of membership of wheat cultivars in each of the seven European countries in each of the two (*K* = 2) and three (*K* = 3) clusters is presented in **Figure 4**. As in FCA and UPGMA analysis, the divergence between subclusters A1 and A2 was moderate in comparison to the split between the two subclusters and the subcluster B as shown by much larger net nucleotide distance (provided by Structure) between A1 and B as well as A2 and B (0.19 and 0.13, respectively) than between A1 and A2 (0.06).

## Discussion

### Genetic Diversity and Population Structure of Croatian Winter Wheat Breeding Pool

Understanding the level and structure of the genetic diversity of a crop is prerequisite for the conservation and efficient use of the available gerpmlasm for plant breeding (Laidò et al., 2013). Further, its monitoring can assist us in the choice of parents with desired alleles and for assessing changes in allelic frequencies (Christiansen et al., 2002). Analysis of Croatian wheat germplasm (89 cultivars) with DArT markers revealed different levels of genetic diversity with an average PIC of 0.30, the expected heterozygosity was 0.375 and effective number of alleles per locus was 1.641. This is within the range of values reported in previous studies on wheat diversity. For example, Stodart et al. (2007) found, when analyzing genetic diversity of 44 wheat landraces from five geographic regions, that 256 DArT markers had a non-adjusted PIC value of 0.43. Raman et al. (2010) analyzed 1057 wheat accesions originating from different geographic regions of the world using 178 DArT loci. The average PIC value was 0.44 with moderate to high levels of diversity. Nei’s diversity indices for modern cultivars were 0.431 and for landrace cultivars 0.427. Zhang et al. (2011) analyzed 111 wheat lines from Northern China and they found for 1637 DArT markers PIC values ranging from 0.03 to 0.50 with an average of 0.40 and Nei’s genetic diversity ranging from 0.11 to 0.50, with an average value of 0.40. White et al. (2008) using DArT markers found that allelic diversity among wheat genomes varied in the sequence A > B > D genomes and for countries was AUS > USA > UK, respectively. Nielsen et al. (2014) found a low proportion of polymorphic DArT markers suggesting a relatively narrow wheat gene pool in Europe. Besides the choice of germplasm, lower than expected PIC values can arise from certain limitations of DArT markers linked with heterozygous and heterogeneous samples as reported by Bolibok-Brkagoszewska et al. (2009).

Our results suggest that only a few genetically similar wheat lines were used for creating genetic variability for breeding purposes and this reflects the historical course of Croatian wheat breeding. Beginning in the 20th century, hybridization was initiated and selections were made from local landraces such as Sirban Prolific and Bankuty wheats. More recently, Italian cultivars including Strampelli’s cultivars (Carlottta Strampelli, Mentana, and San Pastore) were used as progenitors and sources of earliness, high yield capacity, and shorter straw. From the mid 1950s onward, domestic material was crossed mostly with wheat varieties originating from Italy, countries of former Soviet Union, the USA, and France (Denčić, 2001; Brandolini and Vaccino, 2012). The later differences in genetic diversity were introduced by breeders with preferences for different plant ideotypes using diverse sources of germplasm to realize their own breeding objectives.

At *K* = 5 the proportions of membership of PIO cultivars are more evenly distributed among clusters in comparison to those bred by Bc/FAZ leading to a higher total admixture of PIO cultivars based on Shannon’s diversity index. It follows that Bc/FAZ breeding program relies on narrower genetic base that could be efficiently broaden by identifying suitable cultivars belonging to underrepresented subclusters (i.e., A, C, and E) and incorporating them into crossing schemes. However, the admixture attributable to among-cultivar component in case of PIO breeding program is higher than that attributable to within-cultivar component due to the fact that the cultivars classified as representative of different cluster make 26%. It would suggest that the inter-cluster cultivar crossings is likely to result in novel combinations of promising traits.

### Croatian Wheat Germplasm Diversity and Structure within European Context

Combining the genotypic information from the CBP with two additional EBP open-access datasets (Bentley et al., 2014; Nielsen et al., 2014) in the current study allowed a perspective view of the diversity within this regional program.

The results show higher genetic diversity and rare marker frequency in the winter wheat breeding pool from Central Europe (Croatia and Hungary; CE) as compared to the Northern and Western European (NWE) countries (Denmark, France, Germany, Sweden, and UK). In agreement with this, Huang et al. (2002) and Röder et al. (2002), using SSR markers, found the highest diversity in Southern European wheats as compared to those from Northern and Western Europe. The most plausible explanation for the observed levels of genetic diversity and its distribution may be due to the presence of relatively more alleles as a result of breeding practice (Roussel et al., 2005; Hai et al., 2007).

AMOVA showed the most of the genetic diversity was attributable to the differences among cultivars within countries (>80%), which is with the consensus of other reports (Hai et al., 2007). Roussel et al. (2005) and White et al. (2008) found that the geographical factor was more important than the temporal factor in explaining genetic structure, reflecting the wheat breeding practice of longer and more intense selection in NWE than CE, which had different objectives.

When genetic structure of our CBP/EBP wheat panel was analyed the highest Δ*K* value was found at *K* = 2 (**Figure 2B**), but at *K* = 3 there was further separation as well (**Figure 4**). Similar results were reported by Le Couviour et al. (2011) who found clear separation due to geographical specificity at *K* = 2 for wheat varietities originating from UK, Germany and France, but further separation within French cluster was not due to geographic origin or linked to the breeding companies, but rather reinforcing the idea that unique elite variety extensively used as progenitor caused this subdivision.

Further selective changes between NWE and CE wheat breeding pool is due to difference causes by latitude effects, where there is presence of photoperiod sensitive and insensitive alleles causing different adaptation patterns to variable environments.

This provides context for the genetic diversity found and indicates that although the CBP has historically derived parental germplasm from various European and International programs (as above) the diversity retained within it is regionally distinct.

The use of a combined dataset in the current study shows that open-access datasets and common genotyping platforms represent an opportunity to use data-mining to unlock genetic potential. Although useful here for placing the CBP within the context of the wider EBP this approach was limited in this case due to the lack of overlapping DArT markers between the three discrete datasets.

In terms of usefulness in future studies (i.e., for incorporating phenotypic information for mapping) it is also holds limited potential due to the lack of genome-wide coverage, particularly on the D-genome. DArT markers have been superseded by SNP markers which can be applied either singly (e.g., as single marker assays), or in array form. The availability of affordable, high-density markers will add value to future diversity studies, although significant insight into genetic diversity has been possible using DArTs in the current study.

## Conclusion

The current study emphasizes the important contribution made by plant breeders to European wheat genetic diversity as each program tends to represent a pool of regional divergence. This suggests that maintenance of crop diversity as a whole would benefit from an increase in the number of regional breeding programs rather than the consolidation that is often seen, particularly in commercial breeding. As demonstrated here, DArTs showed their efficacy in describing genetic diversity and population structure of the CBP. Likewise, they provided an insight into the distribution of genetic variance within a European context which is mostly held within, rather than between, geographic regions.

## Author Contributions

Conceived and designed the manuscript: DN, AB, ZŠ, MS, and NG. Contributed reagents/materials/analysis tools: GD, DN, ZŠ, RŠ, and KD. Analyzed the data: ZŠ, AB, and RH. Wrote the manuscript: DN, AB, ZŠ, MS, NG, KD, GD, RH, and RŠ.

## Conflict of Interest Statement

The authors declare that the research was conducted in the absence of any commercial or financial relationships that could be construed as a potential conflict of interest.

## References

[B1] Akbari A. M. Wenzl P. Caig V. Carling J. Xia L. Yang S. Y. (2006). Diversity arrays technology (DArT) for high-throughput profiling of the hexaploid wheat genome. *Theor. Appl. Genet.* 113 1409–1420. 10.1007/s00122-006-0365-4 17033786

[B2] Astle W. Balding D. J. (2009). Population structure and cryptic relatedness in genetic association studies. *Statist. Sci.* 24 451–471. 10.1214/09-STS307

[B3] Autrique E. Nachit M. M. Monneveux P. Tanksley S. D. Sorrells M. E. (1996). Genetic diversity in durum wheat based on RFLPs, morphophysiological traits, and coefficient of parentage. *Crop Sci.* 36 735–742. 10.2135/cropsci1996.0011183X003600030036x

[B4] Belkhir K. Borsa P. Chikhi L. Raufaste N. Bonhomme F. (2004). *GENETIX 4.05 logiciel sous Windows TM pour la génétique des populations. Laboratoire Gnome, Populations, Interactions, CNRS UMR 5000*. Montpellier: Université de Montpellier II.

[B5] Bentley A. R. Scutari M. Gosman N. Faure S. Bedford F. Howell P. (2014). Applying association mapping and genomic selection to the dissection of key traits in elite European wheat. *Theor. Appl. Genet.* 127 2619–2633. 10.1007/s00122-014-2403-y 25273129

[B6] Berg E. E. Hamrick J. L. (1997). Quantification of genetic diversity at allozyme loci. *Can. J. Forest. Res.* 27 415–424. 10.1139/x96-195

[B7] Bolibok-Brągoszewska H. Heller-Uszyńnska K. Wenzl P. Uszyńnski G. Killian A. Rakoczy-Trojanowska M. (2009). DArT markers for the rye genome-genetic diversity and mapping. *BMC Genomics* 10:578 10.1186/1471-2164-10-578 PMC2795769 19958552

[B8] Botstein D. White R. L. Sholnick M. David R. W. (1980). Construction of a genetic linkage map in man using restriction fragment length polymorphisms. *Am. J. Hum. Genet.* 32 314–331. 6247908 PMC1686077

[B9] Brandolini A. Vaccino P. (2012). A glimpse into the past: strampelli’s bread wheats legacy. *Genet. Resour. Crop Evol.* 59 839–850. 10.1007/s10722-011-9725-2

[B10] Cardy B. J. Beversdorf W. D. (1984). Identification of soybean cultivars using isoenzyme electrophoresis. *Seed Sci. Technol.* 12 943–954. 10.2135/cropsci2006.06.0434

[B11] Christiansen M. J. Andersen S. B. Ortiz R. (2002). Diversity changes in an intensively bred wheat germplasm during the 20th century. *Mol. Breed.* 9 1–11. 10.1023/A:1019234323372

[B12] Cox T. S. (1998). Deepening the wheat gene pool. *J. Crop Prod.* 1 1–25. 10.1300/J144v01n01_01

[B13] Cox T. S. Kiang Y. T. Gorman M. B. Rodgers D. M. (1985). Relationship between coefficient of parentage and genetic similarity indices in the soybean. *Crop Sci.* 25 529–532. 10.2135/cropsci1985.0011183X002500030023x

[B14] Cox T. S. Murphy J. P. Rodgers D. M. (1986). Changes in genetic diversity in the red winter wheat regions of the United States. *Proc. Natl. Acad. Sci. U.S.A.* 83 5583–5586. 10.1073/pnas.83.15.5583 16593738 PMC386332

[B15] Denčić S. (2001). “Yugoslav wheat pool,” in *The World Wheat Book*, eds Angus W. J. Bonjean B. P. (Paris: Lavoisier Publishing), 377–402.

[B16] Dice L. R. (1945). Measures of the amount of ecologic association between species. *Ecology* 26 297–302. 10.2307/1932409

[B17] Dvorak J. Zhang H. K. (1992). Reconstruction of the phylogeny of the genus Triticum from variation in repeated nucleotide sequences. *Theor. Appl. Genet.* 84 419–429. 10.1007/BF00229502 24203203

[B18] Ehrich D. (2006). AFLPdat: a collection of R functions for convenient handling of AFLP data. *Mol. Ecol.* 6 603–604. 10.1111/j.1471-8286.2006.01380.x

[B19] Ehrich D. Gaudeul M. Assefa A. Koch M. Mummenhoff K. Nemomissa S. (2007). Genetic consequences of Pleistocene range shifts: contrast between the Arctic, the Alps and the East African mountains. *Mol. Ecol.* 16 2542–2559. 10.1111/j.1365-294X.2007.03299.x 17561912

[B20] Evanno G. Regnaut S. Goudet J. (2005). Detecting the number of clusters of individuals using the software STRUCTURE: a simulation study. *Mol. Ecol.* 14 2611–2620. 10.1111/j.1365-294X.2005.02553.x 15969739

[B21] Excoffier L. Laval G. Schneider S. (2005). Arlequin ver. 3.0: an integrated software package for population genetics data analysis. *Evol. Bioinform.* 1 47–50. PMC2658868 19325852

[B22] Excoffier L. Smouse P. E. Quattro J. M. (1992). Analysis of molecular variance inferred from metric distances among DNA haplotypes: application to human mitochondrial DNA restriction data. *Genetics* 131 479–491. 1644282 10.1093/genetics/131.2.479 PMC1205020

[B23] Felsenstein J. (1985). Confidence limits on phylogenesis: an approach using the bootstrap. *Evolution* 39 783–791. 10.1111/j.1558-5646.1985.tb00420.x 28561359

[B24] Guillot G. Carpentier-Skandalis A. (2011). On the informativeness of dominant and co-dominant genetic markers for Bayesian supervised clustering. *Open Statist. Probabil. J.* 3 7–12. 10.2174/1876527001103010007

[B25] Hai L. Wagner C. Friedt W. (2007). Quantitative structure analysis of genetic diversity among spring bread wheats (*Triticum aestivum* L.) from different geographical regions. *Genetica* 130 213–225. 10.1007/s10709-006-9008-6 17048074

[B26] Hammer Ø. Harper D. A. T. Ryan P. D. (2001). PAST: paleontological statistics software package for education and data analysis. *Palaeontol. Electron.* 4:9.

[B27] Harlan J. R. (1972). Genetics of disaster. *J. Environ. Qual.* 1 212–215. 10.2134/jeq1972.13212x

[B28] Huang B. E. George E. W. Forrest K. L. Kilian A. Hayden M. J. Morell M. K. (2012). A multiparent advanced generation inter-cross population for genetic analysis in wheat. *Plant Biotechnol. J.* 7 826–839. 10.1111/j.1467-7652.2012.00702.x 22594629

[B29] Huang X. Q. Börner A. Röder M. S. Ganal M. W. (2002). Assessing genetic diversity of wheat (*Triticum aestivum* L.) germplasm using microsatellite markers. *Theor. Appl. Genet.* 105 699–707. 10.1007/s00122-002-0959-4 12582483

[B30] Jaccoud D. Peng K. Feinstein D. Killian A. (2001). Diversity arrays: a solid state technology for sequence information independent genotyping. *Nucleic Acids Res.* 29:e25. 10.1093/nar/29.4.e25 PMC29632 11160945

[B31] Laidò G. Mangini G. Taranto F. Gadaleta A. Blanco A. Cattivelli L. (2013). Genetic diversity and population structure of tetraploid wheats (*Triticum turgidum* L.) estimated by SSR, DArT and pedigree data. *PLoS ONE* 8:e67280 10.1371/journal.poone.0067280 PMC3694930 23826256

[B32] Landjeva S. Korzun V. Ganeva G. (2006). Evaluation of genetic diversity among Bulgarian winter wheat (*Triticum aestivum* L.) varieties during the period 1925-2003 using microsatellites. *Genet. Resour. Crop Evol.* 53 1605–1614. 10.1007/s10722-005-8718-4

[B33] Le Couviour F. Faure S. Poupard B. Flodrops Y. Dubreuil P. Praud S. (2011). Analysis of genetic structure in a panel of elite wheat varieties and relevance for association mapping. *Theor. Appl. Genet.* 123 715–727. 10.1007/s00122-011-1621-9 21667038

[B34] Lewontin R. C. (1972). The apportionment on human diversity. *Evol. Biol.* 6 381–398. 10.1007/978-1-4684-9063-3_14

[B35] Martin J. M. Blake T. K. Hockett E. A. (1991). Diversity among North American spring barley cultivars based on coefficients of parentage. *Crop Sci.* 31 1131–1137. 10.2135/cropsci1991.0011183X003100050009x

[B36] Mohammadi S. A. Prasanna B. M. (2003). Analysis of genetic diversity in crop plants-salient statistical tools and considerations. *Crop Sci.* 43 1235–1248. 10.2135/cropsci2003.1235

[B37] Morgante M. Salamini F. (2003). From plant genomics to breeding practice. *Curr. Opin. Biotechnol.* 14 214–219. 10.1016/S0958-1669(03)00028-4 12732323

[B38] Nei M. (1973). Analysis of gene diversity in subdivided populations. *Proc. Natl. Acad. Sci. U.S.A.* 70 3321–3323. 10.1073/pnas.70.12.3321 4519626 PMC427228

[B39] Nielsen N. H. Backes G. Stourgaard J. Andersen S. U. Jahoor A. (2014). Genetic diversity and population structure analysis of European hexaploid bread wheat (*Triticum aestivum* L.) *varieties*. *PLoS ONE* 9:e94000 10.1371/journal.pone.0094000 PMC3981729 24718292

[B40] Pritchard J. K. Stephens M. Donnelly P. (2000). Inference of population structure using multilocus genotype data. *Genetics* 155 945–959. 10835412 10.1093/genetics/155.2.945 PMC1461096

[B41] Queen R. A. Gribbon B. M. James C. Jack P. Flavell A. J. (2004). Retrotransposon-based molecular markers for linkage and genetic diversity analysis in wheat. *Mol. Gen. Genomics* 271 91–97. 10.1007/s00438-003-0960-x 14652738

[B42] Raman H. Stodart B. J. Cavanagh C. Mackay M. Morrel M. Milgate A. (2010). Molecular diversity and genetic structure of modern and traditional landrace cultivars of wheat (*Triticum aestivum* L.). *Crop Pasture Sci.* 61 222–229. 10.1071/CP09093

[B43] Röder M. S. Plaschke J. König S. U. Börner A. Sorrells M. E. Tanksley S. D. (1995). Abundance, variability and chromosomal location of microsatellites in wheat. *Mol. Gen. Genet.* 246 327–333. 10.1007/BF00288605 7854317

[B44] Röder M. S. Wendehake K. Korzun V. Bredemeijer G. Laborie D. Bertrand L. (2002). Construction and analysis of a microsatellite-based database of European wheat varieties. *Theor. Appl. Genet.* 106 67–73. 12582872 10.1007/s00122-002-1061-7

[B45] Román B. Rubiales D. Torres A. M. Cubero J. I. Šatović Z. (2001). Genetic diversity in *Orobanche crenata* populations from southern Spain. *Theor. Appl. Genet.* 103 1108–1114. 10.1007/s001220100644

[B46] Roussel V. Leisova L. Exbrayat F. Stehno Z. Balfourier F. (2005). SSR allelic diversity changes in 480 European bread wheat varieties released from 1840 to 2000. *Theor. Appl. Genet.* 111 162–170. 10.1007/s00122-005-2014-8 15887038

[B47] SAS Institute (2004). *SAS/STAT^®^ 9.1 User’s Guide*. Cary, NC: SAS Institute Inc.

[B48] Schönswetter P. Tribsch A. (2005). Vicariance and dispersal in the alpine perenial *Bupleurum stellatum* L. (Apiaceae). *Taxon* 54 725–732. 10.2307/25065429

[B49] Smith J. S. C. Smith O. S. (1987). Associations among inbred lines of maize using electrophoretic, chromatographic, and pedigree data 1. Multivariate and cluster analysis of data from ‘Lancaster Sure Crop’ derived lines. *Theor. Appl. Genet.* 73 654–664. 10.1007/BF00260772 24241187

[B50] Smith J. S. C. Smith O. S. (1988). Associations among inbred lines of maize using electrophoretic, chromatographic, and pedigree data 2. Multivariate and cluster analysis of data from Iowa Stiff Stalk Synthetic derived lines. *Theor. Appl. Genet.* 76 39–44. 10.1007/BF00288829 24231980

[B51] Souza E. Fox P. N. Byerlee D. Skovmand B. (1994). Spring wheat diversity in irrigated areas of two developing countries. *Crop Sci.* 34 774–783. 10.2135/cropsci1994.0011183X003400030031x

[B52] Stodart B. J. Mackay M. C. Raman H. (2007). Assessment of molecular diversity in landraces of bread wheat (*Triticum aestivum* L.) held in an ex situ collection with Diversity Arrays Technology (DArTTM). *Aust. J. Agr. Res.* 58 1174–1182. 10.1071/AR07010

[B53] van de Wouw M. Van Hintum T. Kik C. Van Treuren R. Visser B. (2010). Genetic diversity trends in twentieth century crop cultivars: a meta analysis. *Theor. Appl. Genet.* 120 1241–1252. 10.1007/s00122-009-1252-6 20054521 PMC2839474

[B54] White J. Law J. R. MacKay I. Chalmers K. J. Smith J. S. C. Kilian A. (2008). The genetic diversity of UK, US and Australian cultivars of *Triticum aestivum* measured by DArT markers and considered by genome. *Theor. Appl. Genet.* 116 439–453. 10.1007/s00122-007-0681-3 18060539

[B55] Yu J. Pressoir G. Briggs W. Vroh B. I. Yamasaki M. Doebley J. F. (2006). A unified mixed-model method for association mapping that accounts for multiple levels of relatedness. *Nat. Genet.* 38 203–208. 10.1308/ng1702 16380716

[B56] Zane L. Bargelloni L. Patarnello T. (2002). Strategies for microsatellite isolation: review. *Mol. Ecol.* 11 1–16. 10.1046/j.0962-1083.2001.01418.x 11903900

[B57] Zhang L. Y. Liu D. C. Guo X. L. Yang W. L. Sun J. Z. Wang D. W. (2011). Investigation of genetic diversity and population structure of common wheat cultivars in northern China using DArT markers. *BMC Genet.* 12:42 10.1186/1471-2156-12-42 PMC3114777 21569312

[B58] Zhu C. Gore M. Buckler E. S. Yu J. (2008). Status and prospects of association mapping in plants. *Plant Genome* 1 5–20. 10.3835/plantgenome2008.02.0089

[B59] Zohary D. Harlan J. H. Vardi A. (1969). The wild diploid progenitors of wheat and their breeding value. *Euphytica* 18 58–65.

