# Supplementary material for: Characterizing Croatian Wheat Germplasm Diversity and Structure in a European Context by DArT Markers
Source: Front Plant Sci. 2016 Feb 22;7:184. doi: 10.3389/fpls.2016.00184 (PMC4761793; doi:10.3389/fpls.2016.00184)
Supplement: Supplementary file 1 [file Table_1.DOCX]

Supplementary Material

Characterizing Croatian Wheat Germplasm Diversity and Structure in a European Context

Dario Novoselović, Alison R. Bentley, Ruđer Šimek*, Krešimir Dvojković, Mark E. Sorrels, Nick Grosman, Richard Horsnell, Georg Drezner and Zlatko Šatović

* Correspondence: Ruđer Šimek rsimek@poljinos.hr

**Suplementary Table S1.** Croatian wheat cultivars included in the analysis.

| No. | Cultivar | Program^1^ | Year of release | Pedigree^2^ |
| --- | --- | --- | --- | --- |
| C01 | Adriana | BC | 1988 | Zg 1758-70 / TpR-349 |
| C02 | Afrodita | PIO | 1993 | Zg 772 / Osk. 4.50/1 |
| C03 | Aida | PIO | 2006 | Srpanjka / Rialto |
| C04 | Alka | PIO | 2003 | Osk. 5.140-22-91 / Sana |
| C05 | Aljmašanka | PIO | 1989 | Osk. M.4.13-71-75 /3/ Eph. M68 / Osk. 154-19 // Kavkaz |
| C06 | Ana | PIO | 1988 | Osk. 4. 216-2-76 / Zg 2877-74 |
| C07 | Aura | BC | 1997 | 434 K-4 CM / 7903-93-1 |
| C08 | Barbara | PIO | 1997 | GO 3135 / Žitarka |
| C09 | Bc Antea | BC | 2002 | Zg 213-82 (Sana) / Gala |
| C10 | Bistra | BC | 1977 | Zeka / San Pastore |
| C11 | Danica | PIO | 1993 | Zg5328 / Osk.M.4.13-71-75 /3/ Eph. M68 / Osk. 54-19 // Kavkaz |
| C12 | Dvanaesta | PIO | 1988 | Osk. 4. 216-2-76 / Zg 2877-74 |
| C13 | Edita | PIO | 1998 | Ana / Zrinka |
| C14 | Elza | PIO | 1994 | Slavonija // Zg 2877-74 / Osk.3.328-1-76 /3/ Slavonija |
| C15 | Eva | PIO | 1996 | Slavonija / Skopljanka |
| C16 | Feniks | PIO | 1993 | Slavonija / Skopljanka // Slavonija |
| C17 | Fortuna | PIO | 1991 | Zg 2877-74 / Osk. 4. 216-2-76 |
| C18 | Golubica | PIO | 1998 | Slavonija / Gemini |
| C19 | Hana | PIO | 2000 | GO 3135 / Žitarka |
| C20 | Inga | PIO | 1994 | Slavonija*3 / Skopljanka |
| C21 | Joza | PIO | 1994 | Osk. 3.40-3-81 / MV 23-78 |
| C22 | Julija | PIO | 2000 | F21078-82 / Srpanjka |
| C23 | Kata | PIO | 1997 | Osk. 7.5-4-83 / Žitarka |
| C24 | Katarina | PIO | 2006 | Osk. 5.B.4/1-94 / Osk. 5.140-22-91 |
| C25 | Kiki | PIO | 2002 | Osk.5.36-9-91 / Srpanjka |
| C26 | Klara | PIO | 1998 | Slavonija / Zg 5328-75 |
| C27 | Kleopatra | PIO | 2004 | Sana / Žitarka // Sana |
| C28 | Krušarka | PIO | 1980 | Osk. 7.51-2 // Libellula / Bezostaja-1 |
| C29 | Kuna | FAZ | 1995 | St 563 / Skopljanka |
| C30 | Kupa | BC | 1968 | San Pastore / Heines VII // Gabo/ Pawwee |
| C31 | Lana | BC | 1999 | Zg 583-89 / Kutjevčanka |
| C32 | Lela | PIO | 2006 | Srpanjka / Osk.5.136/8-90 |
| C33 | Liberta | BC | 1997 | M-41-1 / Drina // Zg 167-86 |
| C34 | Lucija | PIO | 2001 | Srpanjka / Kutjevčanka |
| C35 | Magdalen | FAZ | 1995 | Skopljanka /St 563 |
| C36 | Maja | PIO | 1993 | Osk. 4. 159-10 / Slavonija |
| C37 | Manda | PIO | 1994 | Osk. 5.10-2 / Mursa // 2*Slavonija |
| C38 | Marija | BC | 1988 | Zg 4527-68 / Kavkaz // Zg 1971-70 |
| C39 | Martina | PIO | 2000 | Osk. 7.5-3-82 / Srpanjka |
| C40 | Mihelca | BC | 1996 | Zg 1325-78 / SO-1065 |
| C41 | Monika | PIO | 1997 | F21078-82 / Srpanjka |
| C42 | Mura | BC | 1967 | U-18 / Fiorello |
| C43 | Nada | PIO | 1984 | Osječka-20 / Osk. 4.216-2-76 |
| C44 | Neretva | PIO | 1993 | Slavonija / Mačvanka |
| C45 | Nevena | PIO | 2002 | F21078-82 / 2*Osk. 5. 40-51-83 |
| C46 | Nina | BC | 2000 | Zg 213-82 (Sana) / Gala |
| C47 | Njivka | PIO | 1987 | Slavonka / Osk. 5.132-2-74 |
| C48 | Os Elvira | PIO | 2005 | Srpanjka / Kata // Super Žitarka |
| C49 | Osječanka | PIO | 1980 | mutant from Tena (treated with EMS^3^ 1,5 %) |
| C50 | Osječanka 2 | PIO | 1981 | mutant from Tena (treated with EMS 1,5 %) |
| C51 | Osječka 21 | PIO | 1990 | Osk.6.9-1-64/V-188-M |
| C52 | Osječka 22 | PIO | 1990 | Osk.6.9-1-64/V-188-M |
| C53 | Panonija | PIO | 2002 | Ana / Dukat // Ana |
| C54 | Panonka | PIO | 2001 | Zrinka / Osk. 7.5-4-82 |
| C55 | Patria | BC | 1994 | Odesskaya-51 // Zg-IPK-8210 /GK-32-82 |
| C56 | Petra | PIO | 2002 | Osk. 5.104-1-86 // K. 160-86 / Žitarka |
| C57 | Pipi | PIO | 2006 | Soissons / Osk. 6.83-5-91 |
| C58 | Podravina | PIO | 1991 | Zg 5328 / Osk. 4. 216-2-76 |
| C59 | Poljarka | PIO | 1985 | Osk.4.9-5 / Zg-782-72 |
| C60 | Prima | BC | 2001 | Zg 213-82 (Sana) / Gala |
| C61 | Ratarka | PIO | 1985 | Osječka-20 / Osk. 4.216-2-76 |
| C62 | Renata | PIO | 2006 | (Žitarka // (Osk.7.5/4-82 /// Kom.Bg.160/86)) / Srpanjka |
| C63 | Romana | PIO | 2006 | Osk.6.118/4-91 / Osk.4.36/10-90 |
| C64 | Rugvica | BC | 1993 | Zg 169-82 / Sr7i96 // Zg-169-82 /3/ Zg 5373 |
| C65 | Ruža | PIO | 1993 | Osk. 4.150-9 / Osk. 4.8-5-79 |
| C66 | Sana | BC | 1983 | Mura / C.I. 14123 // Zg 2413-72 |
| C67 | Sanja | BC | 1971 | Zg 414-57 / Leonardo |
| C68 | Seka | PIO | 2006 | Srpanjka / Demetra |
| C69 | Senka | PIO | 2003 | Osk. 6.362-2-88 / Edita |
| C70 | Ševa | PIO | 2002 | Aljmašanka / Žitarka // Srpanjka |
| C71 | Sivka | FAZ | 1981 | T. timopheevi derivative 1951 / San Pastore // Bezostaja |
| C72 | Snaša | PIO | 1993 | Skopljanka / Slavonija |
| C73 | Sofija | PIO | 1998 | BH 87-83 / Osk. 3.68-2 |
| C74 | Srpanjka | PIO | 1989 | Osk. 4.50-1 / Zg 2696 |
| C75 | Super Zlatna | BC | 1977 | Zg 5996-66 (Sanja) / Tp 114-1965A // Zg 4730-68(Sanja) |
| C76 | Tena | PIO | 1973 | Libellula / Bezostaja-1 |
| C77 | Teuta | PIO | 2002 | Sana / Žitarka // Sana |
| C78 | Tina | BC | 1993 | Zg 213-82 (Sana) / Gala |
| C79 | Tonka | PIO | 2004 | Osk. 8.37-10-91 / Srpanjka |
| C80 | U-1 | PIO | 1936 | Carlotta Strampelli / Marquis |
| C81 | U-16 | PIO | 1947 | T-25 /3/ K-6 / Sirban Prolific // K-10 / K-65 |
| C82 | Valentina | BC | 1996 | BC-15-85 / ZG-100-95 |
| C83 | Vila | PIO | 2004 | Osk. 6.362-2-88 / Osk. 5.10/7-89 |
| C84 | Vuka | BC | 1964 | Fiorello / U-1 |
| C85 | Zdenka | BC | 1996 | Beauchamp // Kavkaz / Zg 2557-83 |
| C86 | Zlatna Dolina | BC | 1971 | Zg 414-57 / Leonardo |
| C87 | Zlatoklasa | BC | 1978 | Sanja / Tp 114-1965A // Sanja |
| C88 | Zrnka | PIO | 2003 | Osk. 6.30-20 / Slavonka /3/ Eph.M68 / Osk. 154-19 // Kavkaz |
| C89 | Žitarka | PIO | 1985 | Osk.6.30-20 / Slavonka/3/Ephrat M68 /Osk. 154-19//Kavkaz |

^1^Program: PIO - Agricultural Institute Osijek (PIO), BC - Bc Institute for Plant Breeding and Production of Field Crops Production, Zagreb, FAZ - University of Zagreb, Faculty of Agriculture (FAZ)

^2^Pedigree notation according to Purdy, L. H., Loegering, W. Q., Konzak, C. F., Peterson, C. J., and Allan, R. E. (1968). A proposed standard method for illustrating pedigrees of small grain varieties. Crop Sci. 8, 405–406. doi:10.2135/cropsci1968.0011183X000800040002x

^3^Ethyl Methanesulfonate
