# Supplementary material for: Characterizing Croatian Wheat Germplasm Diversity and Structure in a European Context by DArT Markers
Source: Front Plant Sci. 2016 Feb 22;7:184. doi: 10.3389/fpls.2016.00184 (PMC4761793; doi:10.3389/fpls.2016.00184)
Supplement: Supplementary file 2 [file Table_2.DOCX]

Supplementary Material

Characterizing Croatian Wheat Germplasm Diversity and Structure in a European Context

Dario Novoselović, Alison R. Bentley, Ruđer Šimek*, Krešimir Dvojković, Mark E. Sorrels, Nick Grosman, Richard Horsnell, Georg Drezner and Zlatko Šatović

* Correspondence: Ruđer Šimek rsimek@poljinos.hr

**Supplementary Table S2.** Summary of the germplasm used in the current study based on the Croatian (CBP) and European (TG, ED) wheat breeding pools, including number of lines, countries represented, genetic marker type and marker number.

| Panel | N^2^ | Country^3^ | Markers^4^ | N_markers^5^ |
| --- | --- | --- | --- | --- |
| CBP | 89 | 1 | DArT | 1229 |
| TG | 376 | 3 | DArT/SNP | 2712/324 |
| ED | 94 | 16 | DArT | 1849 |
| Combined^1^ | 523 | 7 | DArT | 166 |

^1^Combined dataset based on consolidated data from the three panels minus overlapping lines and skimmed to remove lines and/or markers with ≥10% missing data and countries represented with at least 10 cultivars.

^2^Number of lines in each of the individual panels, and in the combined dataset.

^3^Number of countries represented in each of the individual panels, and in the combined dataset. The complete list of countries in TG and EDP are given in Bentley *et al.* (2014) and Nielsen *et al.* (2014), respectively.

^4^Marker type used for genotyping: DArT (Diversity Array Technology), SNP (single-nucleotide polymorphism).

^5^Number of markers of each type used in each of the individual panels, and in the combined dataset.
