# Supplementary material for: Characterizing Croatian Wheat Germplasm Diversity and Structure in a European Context by DArT Markers
Source: Front Plant Sci. 2016 Feb 22;7:184. doi: 10.3389/fpls.2016.00184 (PMC4761793; doi:10.3389/fpls.2016.00184)
Supplement: Supplementary file 3 [file Table_3.DOCX]

Supplementary Material

Characterizing Croatian Wheat Germplasm Diversity and Structure in a European Context

Dario Novoselović, Alison R. Bentley, Ruđer Šimek*, Krešimir Dvojković, Mark E. Sorrels, Nick Grosman, Richard Horsnell, Georg Drezner and Zlatko Šatović

* Correspondence: Ruđer Šimek rsimek@poljinos.hr

**Supplementary Table S3.** The proportion of membership of 89 Croatian wheat cultivars in each cluster (K = 5) as defined with a model-based clustering method from Pritchard et al. (2000) based on 1,229 DArT markers.

| No. | Cultivar | Program* | Proportion of membership (Q) | | | | | Cluster  membership** |
| --- | --- | --- | --- | --- | --- | --- | --- | --- |
|  |  |  | K5_A | K5_B | K5_C | K5_D | K5_E |  |
| C01 | Adriana | BC | 0.004 | 0.048 | 0.398 | 0.462 | 0.088 | DM |
| C02 | Afrodita | PIO | 0.999 | 0.000 | 0.000 | 0.000 | 0.000 | AR |
| C03 | Aida | PIO | 0.000 | 0.000 | 0.547 | 0.006 | 0.446 | CM |
| C04 | Alka | PIO | 0.001 | 0.001 | 0.493 | 0.505 | 0.000 | DM |
| C05 | Aljmašanka | PIO | 0.134 | 0.132 | 0.586 | 0.148 | 0.000 | CM |
| C06 | Ana | PIO | 0.160 | 0.218 | 0.213 | 0.388 | 0.020 | DM |
| C07 | Aura | BC | 0.175 | 0.362 | 0.007 | 0.439 | 0.017 | DM |
| C08 | Barbara | PIO | 0.573 | 0.413 | 0.002 | 0.011 | 0.001 | AM |
| C09 | Bc Antea | BC | 0.000 | 0.000 | 0.000 | 0.999 | 0.000 | DR |
| C10 | Bistra | BC | 0.122 | 0.604 | 0.096 | 0.177 | 0.001 | BM |
| C11 | Danica | PIO | 0.433 | 0.261 | 0.304 | 0.002 | 0.000 | AM |
| C12 | Dvanaesta | PIO | 0.999 | 0.000 | 0.000 | 0.000 | 0.000 | AR |
| C13 | Edita | PIO | 0.000 | 0.001 | 0.254 | 0.018 | 0.726 | EM |
| C14 | Elza | PIO | 0.970 | 0.023 | 0.000 | 0.006 | 0.001 | AR |
| C15 | Eva | PIO | 0.009 | 0.000 | 0.000 | 0.000 | 0.991 | ER |
| C16 | Feniks | PIO | 0.999 | 0.000 | 0.000 | 0.000 | 0.000 | AR |
| C17 | Fortuna | PIO | 0.610 | 0.287 | 0.001 | 0.102 | 0.000 | AM |
| C18 | Golubica | PIO | 0.812 | 0.001 | 0.111 | 0.073 | 0.004 | AG |
| C19 | Hana | PIO | 0.490 | 0.507 | 0.001 | 0.000 | 0.001 | BM |
| C20 | Inga | PIO | 0.999 | 0.000 | 0.000 | 0.000 | 0.000 | AR |
| C21 | Joza | PIO | 0.139 | 0.179 | 0.336 | 0.346 | 0.001 | DM |
| C22 | Julija | PIO | 0.000 | 0.057 | 0.548 | 0.000 | 0.394 | CM |
| C23 | Kata | PIO | 0.368 | 0.592 | 0.001 | 0.038 | 0.001 | BM |
| C24 | Katarina | PIO | 0.001 | 0.000 | 0.313 | 0.034 | 0.652 | EM |
| C25 | Kiki | PIO | 0.556 | 0.000 | 0.440 | 0.003 | 0.000 | AM |
| C26 | Klara | PIO | 0.488 | 0.511 | 0.001 | 0.000 | 0.001 | BM |
| C27 | Kleopatra | PIO | 0.000 | 0.001 | 0.001 | 0.998 | 0.000 | DR |
| C28 | Krušarka | PIO | 0.446 | 0.527 | 0.001 | 0.002 | 0.024 | BM |
| C29 | Kuna | FAZ | 0.000 | 0.017 | 0.173 | 0.008 | 0.802 | EG |
| C30 | Kupa | BC | 0.002 | 0.701 | 0.001 | 0.296 | 0.000 | BM |
| C31 | Lana | BC | 0.228 | 0.552 | 0.216 | 0.003 | 0.001 | BM |
| C32 | Lela | PIO | 0.379 | 0.204 | 0.416 | 0.001 | 0.000 | CM |
| C33 | Liberta | BC | 0.001 | 0.000 | 0.000 | 0.998 | 0.000 | DR |
| C34 | Lucija | PIO | 0.081 | 0.162 | 0.710 | 0.047 | 0.000 | CM |
| C35 | Magdalen | FAZ | 0.000 | 0.105 | 0.000 | 0.000 | 0.894 | EG |
| C36 | Maja | PIO | 0.000 | 0.000 | 0.000 | 0.000 | 0.999 | ER |
| C37 | Manda | PIO | 0.656 | 0.142 | 0.102 | 0.099 | 0.000 | AM |
| C38 | Marija | BC | 0.000 | 0.212 | 0.002 | 0.121 | 0.665 | EM |
| C39 | Martina | PIO | 0.169 | 0.396 | 0.416 | 0.015 | 0.005 | CM |
| C40 | Mihelca | BC | 0.203 | 0.647 | 0.022 | 0.128 | 0.001 | BM |
| C41 | Monika | PIO | 0.000 | 0.000 | 0.569 | 0.018 | 0.412 | CM |
| C42 | Mura | BC | 0.158 | 0.727 | 0.000 | 0.114 | 0.001 | BM |
| C43 | Nada | PIO | 0.998 | 0.000 | 0.000 | 0.000 | 0.000 | AR |
| C44 | Neretva | PIO | 0.239 | 0.000 | 0.000 | 0.000 | 0.760 | EG |
| C45 | Nevena | PIO | 0.127 | 0.481 | 0.110 | 0.279 | 0.003 | BM |
| C46 | Nina | BC | 0.001 | 0.000 | 0.001 | 0.998 | 0.000 | DR |
| C47 | Njivka | PIO | 0.242 | 0.755 | 0.000 | 0.003 | 0.000 | BG |
| C48 | Os Elvira | PIO | 0.663 | 0.269 | 0.067 | 0.001 | 0.000 | AM |
| C49 | Osječanka | PIO | 0.001 | 0.999 | 0.000 | 0.000 | 0.000 | BR |
| C50 | Osječanka 2 | PIO | 0.001 | 0.999 | 0.000 | 0.000 | 0.000 | BR |
| C51 | Osječka 21 | PIO | 0.367 | 0.187 | 0.443 | 0.003 | 0.000 | CM |
| C52 | Osječka 22 | PIO | 0.005 | 0.161 | 0.147 | 0.687 | 0.000 | DM |
| C53 | Panonija | PIO | 0.177 | 0.476 | 0.130 | 0.216 | 0.002 | BM |
| C54 | Panonka | PIO | 0.271 | 0.264 | 0.453 | 0.001 | 0.011 | CM |
| C55 | Patria | BC | 0.101 | 0.575 | 0.195 | 0.120 | 0.008 | BM |
| C56 | Petra | PIO | 0.000 | 0.001 | 0.000 | 0.000 | 0.999 | ER |
| C57 | Pipi | PIO | 0.359 | 0.398 | 0.001 | 0.241 | 0.001 | BM |
| C58 | Podravina | PIO | 0.344 | 0.278 | 0.337 | 0.040 | 0.002 | AM |
| C59 | Poljarka | PIO | 0.247 | 0.748 | 0.000 | 0.004 | 0.000 | BM |
| C60 | Prima | BC | 0.001 | 0.000 | 0.000 | 0.998 | 0.000 | DR |
| C61 | Ratarka | PIO | 0.999 | 0.000 | 0.000 | 0.000 | 0.000 | AR |
| C62 | Renata | PIO | 0.183 | 0.180 | 0.499 | 0.138 | 0.001 | CM |
| C63 | Romana | PIO | 0.287 | 0.000 | 0.712 | 0.001 | 0.000 | CM |
| C64 | Rugvica | BC | 0.009 | 0.389 | 0.147 | 0.451 | 0.004 | DM |
| C65 | Ruža | PIO | 0.222 | 0.000 | 0.000 | 0.000 | 0.777 | EG |
| C66 | Sana | BC | 0.000 | 0.000 | 0.000 | 0.999 | 0.000 | DR |
| C67 | Sanja | BC | 0.054 | 0.339 | 0.001 | 0.606 | 0.000 | DM |
| C68 | Seka | PIO | 0.042 | 0.007 | 0.776 | 0.173 | 0.001 | CG |
| C69 | Senka | PIO | 0.241 | 0.375 | 0.229 | 0.154 | 0.001 | BM |
| C70 | Ševa | PIO | 0.000 | 0.000 | 0.454 | 0.000 | 0.545 | EM |
| C71 | Sivka | FAZ | 0.296 | 0.499 | 0.075 | 0.117 | 0.014 | BM |
| C72 | Snaša | PIO | 0.000 | 0.000 | 0.000 | 0.000 | 0.999 | ER |
| C73 | Sofija | PIO | 0.293 | 0.408 | 0.101 | 0.197 | 0.001 | BM |
| C74 | Srpanjka | PIO | 0.000 | 0.000 | 0.999 | 0.000 | 0.000 | CR |
| C75 | Super Zlatna | BC | 0.076 | 0.290 | 0.012 | 0.621 | 0.001 | DM |
| C76 | Tena | PIO | 0.000 | 0.999 | 0.000 | 0.000 | 0.000 | BR |
| C77 | Teuta | PIO | 0.001 | 0.001 | 0.044 | 0.954 | 0.000 | DR |
| C78 | Tina | BC | 0.001 | 0.000 | 0.000 | 0.999 | 0.000 | DR |
| C79 | Tonka | PIO | 0.081 | 0.118 | 0.800 | 0.000 | 0.000 | CG |
| C80 | U-1 | PIO | 0.033 | 0.195 | 0.771 | 0.001 | 0.000 | CG |
| C81 | U-16 | PIO | 0.010 | 0.682 | 0.001 | 0.188 | 0.120 | BM |
| C82 | Valentina | BC | 0.168 | 0.567 | 0.001 | 0.263 | 0.001 | BM |
| C83 | Vila | PIO | 0.462 | 0.446 | 0.000 | 0.092 | 0.000 | AM |
| C84 | Vuka | BC | 0.002 | 0.685 | 0.001 | 0.311 | 0.000 | BM |
| C85 | Zdenka | BC | 0.308 | 0.198 | 0.003 | 0.491 | 0.000 | DM |
| C86 | Zlatna Dolina | BC | 0.345 | 0.006 | 0.001 | 0.647 | 0.000 | DM |
| C87 | Zlatoklasa | BC | 0.164 | 0.011 | 0.168 | 0.656 | 0.000 | DM |
| C88 | Zrnka | PIO | 0.000 | 0.001 | 0.395 | 0.012 | 0.591 | EM |
| C89 | Žitarka | PIO | 0.065 | 0.285 | 0.000 | 0.000 | 0.649 | EM |

^1^Program: PIO - Agricultural Institute Osijek (PIO), BC - Bc Institute for Plant Breeding and Production of Field Crops Production, Zagreb, FAZ - University of Zagreb, Faculty of Agriculture (FAZ)

^2^First letter designates the cluster as defined with a model-based clustering method from Pritchard et al. (2000) while the second classifies cultivars as R - representatives of the cluster (those having more than 90% of their genome estimated to belong to a cluster), G - cultivars that belong to the cluster (membership probabilities between 75 and 90%) or M - mixed (with membership probabilities < 75% for all clusters)
