# Supplementary material for: Characterizing Croatian Wheat Germplasm Diversity and Structure in a European Context by DArT Markers
Source: Front Plant Sci. 2016 Feb 22;7:184. doi: 10.3389/fpls.2016.00184 (PMC4761793; doi:10.3389/fpls.2016.00184)
Supplement: Supplementary file 4 [file Table_4.DOCX]

Supplementary Material

Characterizing Croatian Wheat Germplasm Diversity and Structure in a European Context

Dario Novoselović, Alison R. Bentley, Ruđer Šimek*, Krešimir Dvojković, Mark E. Sorrels, Nick Grosman, Richard Horsnell, Georg Drezner and Zlatko Šatović

* Correspondence: Ruđer Šimek rsimek@poljinos.hr

**Supplementary Table S4.** Distribution of the 166 common DArT markers used to generate the combined CBP/EBP dataset across the wheat genome.

|  | Genome | | | |  |
| --- | --- | --- | --- | --- | --- |
| Chromosome | A | B | | D | Total by group |
| 1 | 12 | 10 | | 4 | 26 |
| 2 | 6 | 13 | | 2 | 21 |
| 3 | 8 | 14 | | 3 | 25 |
| 4 | 11 | 3 | | 0 | 14 |
| 5 | 2 | 12 | | 1 | 15 |
| 6 | 14 | 16 | | 0 | 30 |
| 7 | 11 | 12 | | 5 | 28 |
| Total by genome | 64 | 80 | | 15 | 159 |
| Unmapped |  |  |  | | 7 |
| TOTAL |  |  |  | | 166 |
