# Supplementary material for: Characterizing Croatian Wheat Germplasm Diversity and Structure in a European Context by DArT Markers
Source: Front Plant Sci. 2016 Feb 22;7:184. doi: 10.3389/fpls.2016.00184 (PMC4761793; doi:10.3389/fpls.2016.00184)
Supplement: Supplementary file 5 [file Image_1.PDF]

Supplementary Material

Characterizing Croatian Wheat Germplasm Diversity and Structure in a European Context

Dario Novoselović, Alison R. Bentley, Ruđer Šimek*, Krešimir Dvojković, Mark E. Sorrels, Nick Grosman, Richard Horsnell, Georg Drezner and Zlatko Šatović

*** Correspondence: Ruđer Šimek rsimek@poljinos.hr**

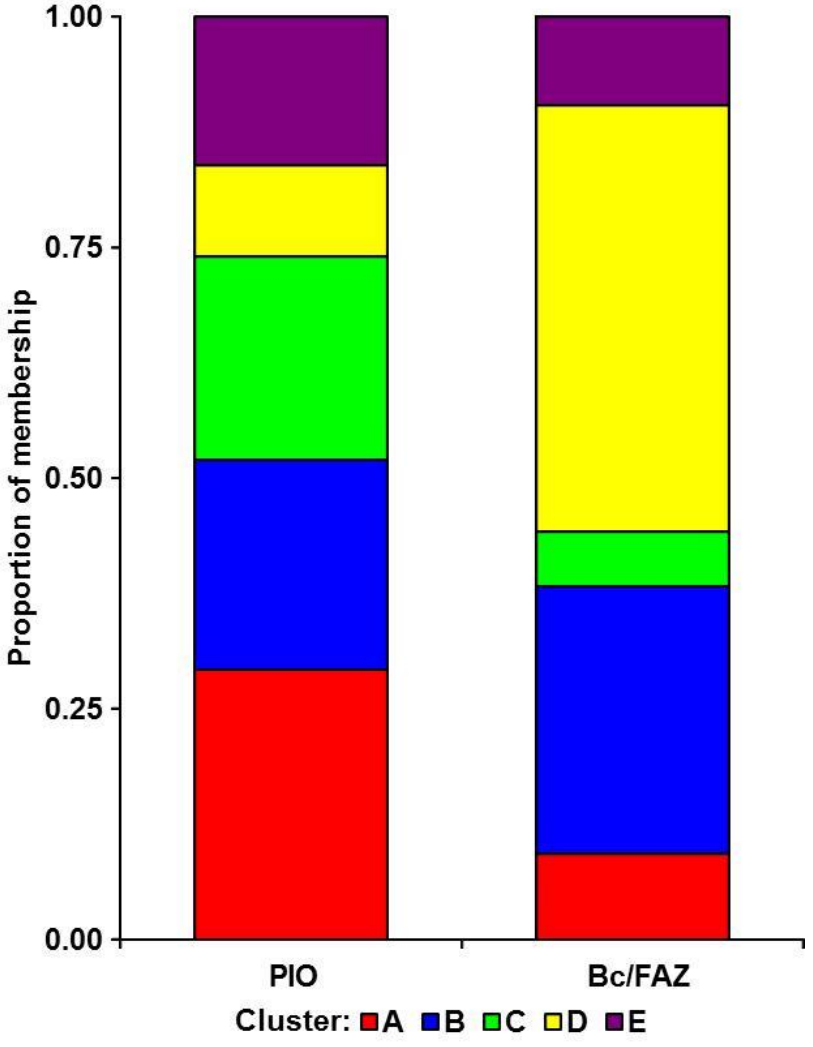

Supplementary Figure S1. Mean proportion of membership of Croatian wheat cultivars bred by the Agricultural Institute Osijek (PIO) and the Bc Institute for Plant Breeding and Production of field crops or the University of Zagreb, Faculty of Agriculture (Bc/FAZ) in each cluster at K = 5 as defined with a model-based clustering method from Pritchard et al. (2000) based on 1,229 DArT markers.
